# MR sequence design to account for nonideal gradient performance

**DOI:** 10.1002/mrm.70093

**Published:** 2025-09-22

**Authors:** Daniel J. West, Felix Glang, Jonathan Endres, David Leitão, Moritz Zaiss, Joseph V. Hajnal, Shaihan J. Malik

**Affiliations:** ^1^ Imaging Physics & Engineering Research Department School of Biomedical Engineering & Imaging Sciences, King's College London London UK; ^2^ Max‐Planck‐Institut fur Biologische Kybernetik Magnetic Resonance Center Tübingen Germany; ^3^ Institute of Neuroradiology Erlangen University Hospital Institute of Neuroradiology Erlangen Germany; ^4^ Department of Artificial Intelligence in Biomedical Engineering Friedrich‐Alexander‐Universität Erlangen‐Nurnberg Erlangen Germany; ^5^ Early Life Imaging Research Department School of Biomedical Engineering & Imaging Sciences, King's College London London UK

**Keywords:** eddy currents, gradient imperfections, gradient system transfer function, pre‐emphasis

## Abstract

**Purpose:**

MRI systems are traditionally engineered to produce close to idealized performance, enabling a simplified pulse sequence design philosophy. An example of this is control of eddy currents produced by gradient fields; usually these are compensated by pre‐emphasizing demanded waveforms. This process typically happens invisibly to the pulse sequence designer, allowing them to assume achieved gradient waveforms will be as desired. Although convenient, this requires system specifications exposed to the end user to be substantially down‐rated, as pre‐emphasis adds an extra overhead to the waveforms. This strategy is undesirable for lower performance or resource‐limited hardware. Instead, we propose an optimization‐based method to design precompensated gradient waveforms that (i) explicitly respect hardware constraints and (ii) improve imaging performance by correcting k‐space samples directly.

**Methods:**

Gradient waveforms are numerically optimized by including a model for system imperfections. This is investigated in simulation using an exponential eddy current model, then experimentally using an empirical gradient system transfer function on a 7T MRI system.

**Results:**

Our proposed method discovers solutions that produce negligible reconstruction errors while satisfying gradient system limits, even when classic pre‐emphasis produces infeasible results. Substantial reduction in ghosting artifacts from echo‐planar imaging was observed, including an average reduction of 77% in ghost amplitude in phantoms.

**Conclusions:**

This work demonstrates numerical optimization of gradient waveforms, yielding substantially improved image quality when given a model for system imperfections. Although the method as implemented has limited flexibility, it could enable more efficient hardware use and may prove particularly important for maximizing performance of lower‐cost systems.

## 1 INTRODUCTION

MRI system hardware is traditionally engineered to produce close to idealized performance, enabling a simplified pulse‐sequence design philosophy in which the designer considers that the system will produce fields as demanded. A key requirement is that subsystems, particularly the radiofrequency transmit and gradient systems, (i) can operate independently from one time period to the next, (ii) are precisely synchronized in time, and (iii) respond linearly. A challenge to this ideal are gradient‐induced eddy currents^1^, ^2^, ^3^, ^4^ (due to rapidly changing magnetic fields applied during MR acquisitions), gradient time delays, and mechanical resonances, which can be characterized by a gradient system transfer function (GSTF).

To retain the idealized performance philosophy, these effects are often compensated by “pre‐emphasizing” demanded waveforms after sequence definition but before amplification by gradient power amplifiers (GPAs). Although convenient, this necessitates enforcement of more stringent limits on hardware performance than the hardware can handle, as pre‐emphasis adds an extra overhead to the waveforms. The idealized performance philosophy is effective, as evidenced by its universal adoption, but other methods that acknowledge and embrace true system performance are now feasible.

Assuming that an MR system is linear time‐invariant, GSTFs are often applied during image reconstruction to perform post hoc correction for gradient imperfections. Pre‐acquisition correction strategies based on inversion of the GSTF have previously been demonstrated,^5^, ^6^, ^7^ where demanded gradient waveforms are pre‐emphasized using frequency domain division by the GSTF. This is equivalent to more standard pre‐emphasis methods but with the usual time domain decaying exponential model^8^ replaced by a GSTF. Frequency domain division is, however, susceptible to zeros and GSTF inaccuracies, and cannot be done in a way that respects scanner hardware limits.

Both standard pre‐emphasis and frequency domain correction lead to increased demanded slew rates, to counteract the smoothing effect of eddy currents. To respect hardware constraints, a typical solution “down‐rates” initial gradient waveforms by designing them for a lower maximum slew rate and peak amplitude, such that once corrected, they remain within the system's actual capability. This approach was taken by Vannesjo et al.^5^ for GSTF‐based correction; although practical, the system's resources are not optimally used. For scenarios with significant eddy currents, this results in substantial performance loss.

We propose an optimization‐based method that designs precompensated gradient waveforms by considering a system distortion model and explicitly respects hardware constraints with a view to maximizing imaging performance. To gain more flexibility, rather than correcting time domain waveforms, we instead correct their zeroth‐order moment (i.e., the k‐space that they define), as this is ultimately the quantity of interest. The application of this method to echo‐planar imaging (EPI) sequences is explored using an exponential eddy current model and empirically measured GSTF (accounting for overall gradient performance).

## 2 THEORY

### 2.1 Gradient system models and pre‐emphasis

To model gradient imperfections, we distinguish between demanded control inputs and realized fields. We denote demanded waveforms as g(t), their temporal derivative as $\dot{g}\left(t\right)$, and realized waveforms as G(t). To enable comparison with conventional sequence design strategies, both are expressed in the same units (mT/m), although g(t) would be realized as a current (or voltage) signal sent to GPAs. A common approach for quantifying (nonideal) gradient performance is the following exponential model^9^:

(1) $$G\left(t\right)=f\left\{g\left(t\right)\right\}=g\left(t\right)-\dot{g}\left(t\right)⊗\theta \left(t\right)\sum_{n}\alpha_{n}\exp\left(-\frac{t}{\tau_{n}}\right)$$

where $\alpha_{n}$ and $\tau_{n}$ are amplitude and time constants; θ(t) is a unit step function; and ⊗ represents a convolution. To a first‐order approximation, demanded waveforms can be pre‐emphasized using^8^:

(2) $$\begin{array}{c} g^{\mathrm{PRE}}\left(t\right)=g\left(t\right)+\dot{g}\left(t\right)⊗\theta \left(t\right)\sum_{n}{\tilde{\alpha }}_{n}\exp\left(-\frac{t}{{\tilde{\tau }}_{n}}\right) \end{array}$$

where ${\tilde{\alpha }}_{n}$ and ${\tilde{\tau }}_{n}$ are numerically optimized such that $f\left\{g^{\mathrm{PRE}}\left(t\right)\right\}\approx g\left(t\right)$ (i.e., ${\tilde{\alpha }}_{n}$ and ${\tilde{\tau }}_{n}$ deviate from true values of $\alpha_{n}$ and $\tau_{n}$ in the forward model).

Alternatively, gradient system response can be described using a GSTF, H(ω), assuming linear time‐invariance:

(3A) $$\begin{array}{c} \tilde{G}\left(\omega \right)=H\left(\omega \right)\tilde{g}\left(\omega \right) \end{array}$$

where ω is temporal frequency, and variables with tildes are frequency domain representations. This is written as a matrix to include cross‐terms. The relationship can also be expressed in the time domain as a convolution:

(3B) $$\begin{array}{c} G=h\left(t\right)⊗g\left(t\right) \end{array}$$

(3C) $$\begin{array}{c} \left[\begin{array}{c} G_{x}\left(t\right)\\ G_{y}\left(t\right)\\ G_{z}\left(t\right) \end{array}\right]=\left[\begin{array}{ccc} h_{\mathrm{xx}}\left(t\right) & h_{\mathrm{yx}}\left(t\right) & h_{\mathrm{zx}}\left(t\right)\\ h_{\mathrm{xy}}\left(t\right) & h_{\mathrm{yy}}\left(t\right) & h_{\mathrm{zy}}\left(t\right)\\ h_{\mathrm{xz}}\left(t\right) & h_{\mathrm{yz}}\left(t\right) & h_{\mathrm{zz}}\left(t\right) \end{array}\right]⊗\left[\begin{array}{c} g_{x}\left(t\right)\\ g_{y}\left(t\right)\\ g_{z}\left(t\right) \end{array}\right] \end{array}$$

where h(t) is the time domain representation of the GSTF (i.e., the gradient impulse response function [GIRF]). Cross‐terms are written explicitly here. The GSTF can be measured empirically for an MR scanner including any pre‐emphasis correction applied invisibly to the user. In principle, it may be extended to include both zero‐order and higher‐order terms,^10^ but these were not explored in this work for simplicity. Preliminary exploration indicated that first‐order terms were the dominant source of error for sequences studied here. Additionally, the GSTF can be used for pre‐emphasis by frequency domain division^6^:

(4) $$\begin{array}{c} {\tilde{g}}^{\mathrm{PRE}}\left(\omega \right)=H^{-1}\left(\omega \right)\tilde{g}\left(\omega \right) \end{array}$$

### 2.2 Optimization framework

Pre‐emphasis described by Eqs. (2) and (4) aims to produce realized gradients G(t) that closely match the desired, ideal behavior. Here, we instead optimize g(t) to produce k‐space locations $k_{i}\equiv \gamma \int_{0}^{t_{i}}G\left(t^{\prime }\right)\;{\mathrm{dt}}^{\prime }$ at sample times $t_{i}$ that align with desired k‐space sample locations $k_{0}$. This is achieved by optimization as follows:

(5A) $$\begin{array}{c} g_{\mathrm{op}}\left(t\right)=\text{argmin}\left\{\sum_{i=1}^{N_{k}}{\left\|k_{i}-k_{0,i}\right\|}^{2}\right\} \end{array}$$

subject to

(5B) $$\begin{array}{c} \left|g_{\mathrm{op}}\left(t\right)\right|\leq g_{\max} \end{array}$$

(5C) $$\begin{array}{c} \left|{\dot{g}}_{\mathrm{op}}\left(t\right)\right|\leq s_{\max} \end{array}$$

Where $g_{\max}$ and $s_{\max}$ are limits on demanded gradient amplitude and slew rate, respectively. In practice, these constraints are realized by adding penalty terms to the optimization as follows:

(6) $$\begin{array}{cc} g_{\mathrm{op}}\left(t\right) & =\text{argmin}\left\{\sum_{i=1}^{N_{k}}{\left\|k_{i}-k_{0,i}\right\|}^{2}+w_{g}\sum_{i=1}^{N_{t}}\max\left(0,\left|g\left(t_{i}\right)\right|-g_{\max}\right)\right.\\ & \;\left.+w_{\dot{g}}\sum_{i=1}^{N_{t}}\max\left(0,\left|\dot{g}\left(t_{i}\right)\right|-s_{\max}\right)\;\right\} \end{array}$$

where $N_{t}$ is the number of timepoints; max operations enforce penalty terms that are only active when the constraints are violated; and w are tunable weighting factors.

## 3 METHODS

The proposed method was implemented as an extension to the MR‐zero framework^11^, ^12^ and tested for several EPI sequence variants using simulated eddy currents (assuming an exponential model) and experimentally on a 7T MR scanner (Magnetom Terra; Siemens Healthineers, Forchheim, Germany) using a measured GSTF. The response function was measured using a modified thin‐slice method with in‐plane spatial encoding to capture cross‐terms, as proposed by Rahmer et al.,^13^ using a spherical water phantom and a chirped test waveform.

Optimizations were run using the Adam^14^ algorithm, taking advantage of pytorch/autograd to propagate derivatives through the loss function. Because the gradient distortion model (exponential or GSTF) is not a function of the gradient sample points being optimized, it can be treated as a constant from the perspective of propagation of derivatives. MR‐zero with phase distribution graph^15^ signal simulation was used to predict images obtained from pre‐optimization and post‐optimization sequences with a two‐dimensional numerical brain phantom.^16^ Optimized sequences were tested on both a spherical water phantom and healthy volunteer (female, age 25) who gave written consent under local ethical approval (HR‐18/19‐8700).

### 3.1 Example EPI sequences

Four variants of a single‐slice two‐dimensional EPI sequence were explored: single‐shot using acceleration factors *R* = 1, 2, and 3, and multishot (16 shots, repetition time = 5 s, *R* = 1). The sequence selection is arbitrary, but use of these different acceleration factors and shot lengths changes the frequency content, making potential GSTF‐mediated errors different. All sequences had a 250‐mm field of view; the single‐shot *R* = 1 sequence had matrix size = 96; *R* = 2 and multishot had matrix size = 128; and *R* = 3 had matrix size = 129. The multishot sequence had echo time (TE) = 13 ms. Partial Fourier sampling was used to maintain similar TEs for all single‐shot scans (TE = 33.8/33.6/36.6 ms for *R* = 1/2/3 cases, respectively, with partial Fourier factors 73/96 for *R* = 1 and 102/128 for *R* = 2). An additional version of the *R* = 1 sequence (termed shorter TE) was optimized using a shorter interecho spacing (1.05 ms instead of 1.07 ms); the nominal equivalent exceeds the scanner slew rate limit and so was not acquired.

Images were reconstructed using an inverse Fourier transform (iFT) and basic GRAPPA implementation^17^, ^18^ where data were under‐sampled. For these cases, a FLASH scan with matrix size = 128 × 128 was used to obtain calibration data, with GRAPPA kernel size [kx, ky]= [5, 9]. Partial Fourier‐induced Gibbs ringing was reduced using a smooth‐step filter. No phase corrections were made for ghost correction of EPI data.

### 3.2 Simulated experiments with exponential eddy current model

Single‐shot (*R* = 1) EPI optimizations assumed a scanner‐typical eddy current term (τ = 50 μs). Its scaling parameter was adjusted to produce a significant but not unrealistic perturbation (α = 5e‐6). Optimization of the 17 040 gradient samples was performed on a 20(40) × Intel Xeon Silver 4210 2.20‐GHz CPU, 251GB RAM, 32GB NVIDIA Tesla V100 GPU (NVIDIA, Santa Clara, CA, USA), with realistic scanner hardware limits and a learning rate of 5e‐5. The optimization result was compared with that obtained using pre‐emphasis (with separately optimized eddy current properties: $\tilde{\alpha }$ = 1e‐5 and $\tilde{\tau }$ = 22.2 μs) using normalized root‐mean‐square (RMS) error of pixel intensities calculated with respect to the target image.

### 3.3 Physical experiments using MR system with empirical GSTF

Optimizations of all four EPI variants were completed using Eq. (3B) as the hardware model (only first‐order terms were considered). Plots of measured terms for different physical axes are shown in Figure S1. Scanner amplitude and slew rate limits of $g_{\max}$ = 72 mT/m and $s_{\max}$ = 180 mT/m/ms were enforced during optimization. Optimization solutions are compared with those obtained when inverting the GSTF (Eq. [4]) using normalized RMS error. GSTF‐based pre‐emphasis was computed using only self‐terms and a Tukey filter (45‐kHz cut‐off frequency, 0.3 transition width) to suppress noise amplification due to zeroes at high GSTF frequencies.

Because the GSTF was measured using the scanner's own pre‐emphasis correction, it characterizes the system response when operating under normal conditions. All EPI experiments were run under these conditions; hence, both GSTF‐based pre‐emphasis and optimized solutions should be understood as additional corrections, beyond any existing gradient corrections the scanner uses.

EPI gradient waveforms were discretized on a time grid matching the gradient raster time (10 μs). A learning rate of 5e‐6 was used with 50 000 iterations for all single‐shot cases (taking ˜40 mins with ˜0.05 s per iteration) and 20 000 iterations for multishot cases. All sequences were implemented using *Pulseq*.^19^, ^20^ Ghosting artifacts were quantified by taking the difference between RMS signal outside the object in phase‐encode and frequency‐encode directions, normalized to RMS signal in the phantom.

## 4 RESULTS

### 4.1 Simulations using exponential eddy current model

**FIGURE 1:**
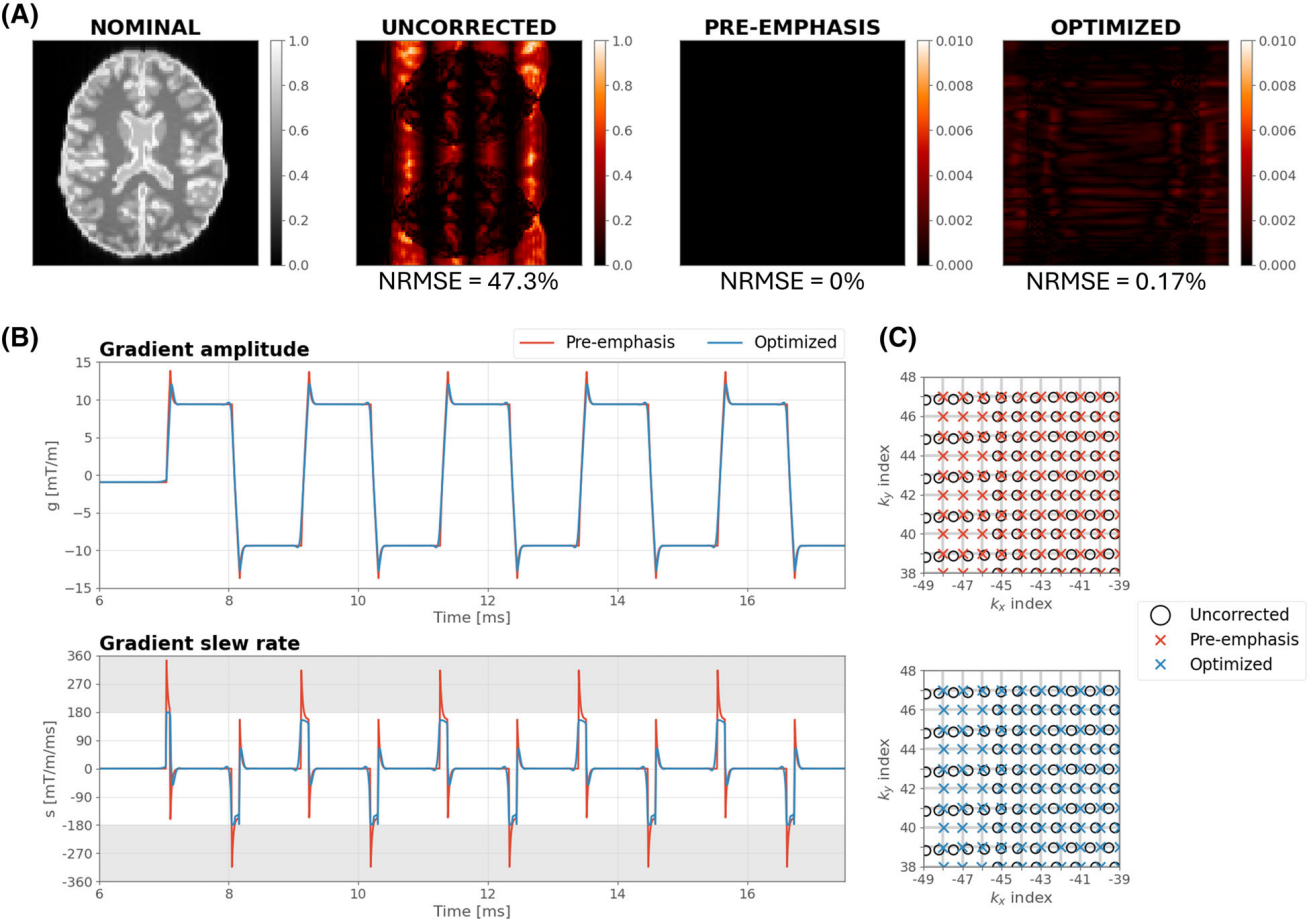
Comparison of our optimization results to standard pre‐emphasis when using a simulated exponential eddy current model with *τ* = 50 μs. (A) The nominal image reconstruction (no gradient errors) alongside corresponding differences when eddy currents are included before correction, after pre‐emphasis, and after optimization (normalized root‐mean‐square error [NRMSE] displayed underneath). (B) Demanded gradients and corresponding slew rates are plotted over a zoomed timescale to highlight waveform features, with k‐space locations shown in (C) (*gray lines correspond to the desired k‐space grid*). Optimization finds a solution with almost perfectly restored k‐space samples while satisfying scanner hardware constraints. Pre‐emphasis also corrects the k‐space locations but exceeds slew rate constraints (*forbidden regions are shaded gray in gradient slew rate plot*).

Figure 1 shows optimized gradient waveforms compared with the standard pre‐emphasis solution in simulation. Both show similar characteristics, but the optimized solution does not violate the defined slew rate limit (180 mT/m/ms). Sample locations are restored to a regular Cartesian grid using either approach, as shown in the plotted k‐space portions, with negligible image reconstruction error (< 0.2%) for both.

### 4.2 Simulations using GSTF model

**FIGURE 2:**
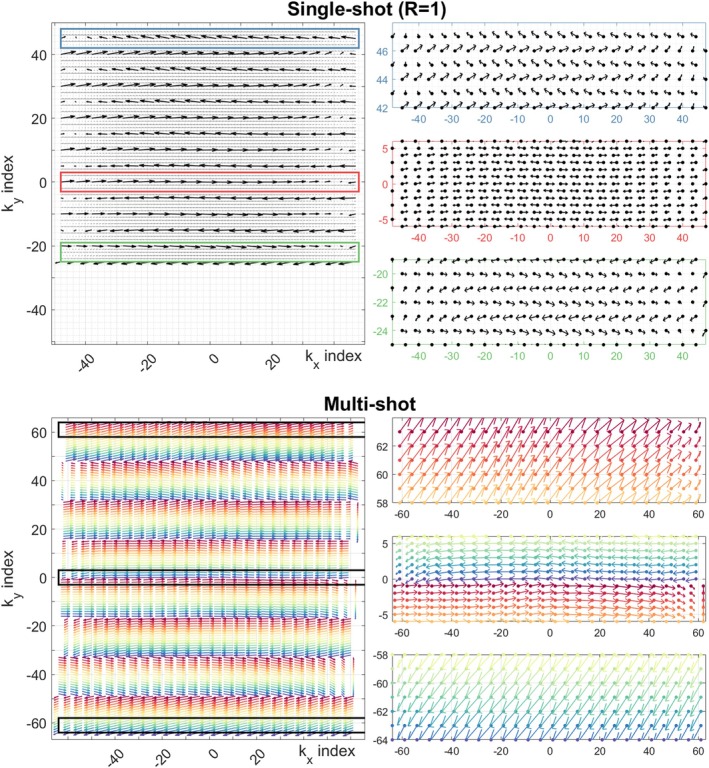
k‐space displacements indicated by arrows pointing from nominal demanded locations to realized locations for entire k‐spaces and zoomed k‐space regions to visualize displacement more clearly, with dots indicating nominal demanded locations. As arrow sizes and directions are spatially inconsistent, post hoc phase shifts would be unsuccessful in mitigating image imperfections. Down‐sampling of arrows is performed for clarity. Multishot arrows are colored according to shot number. Partial Fourier is visible in the top LHS for the single‐shot example.

Figure 2 illustrates k‐space displacements caused by gradient imperfections as modeled by the GSTF for examples of single‐shot and multishot EPI sequences. Odd and even lines/shots are not just offset from one another; different degrees of displacement are seen throughout k‐space. The proposed optimization and GSTF‐based pre‐emphasis aim to eliminate these shifts.

**FIGURE 3:**
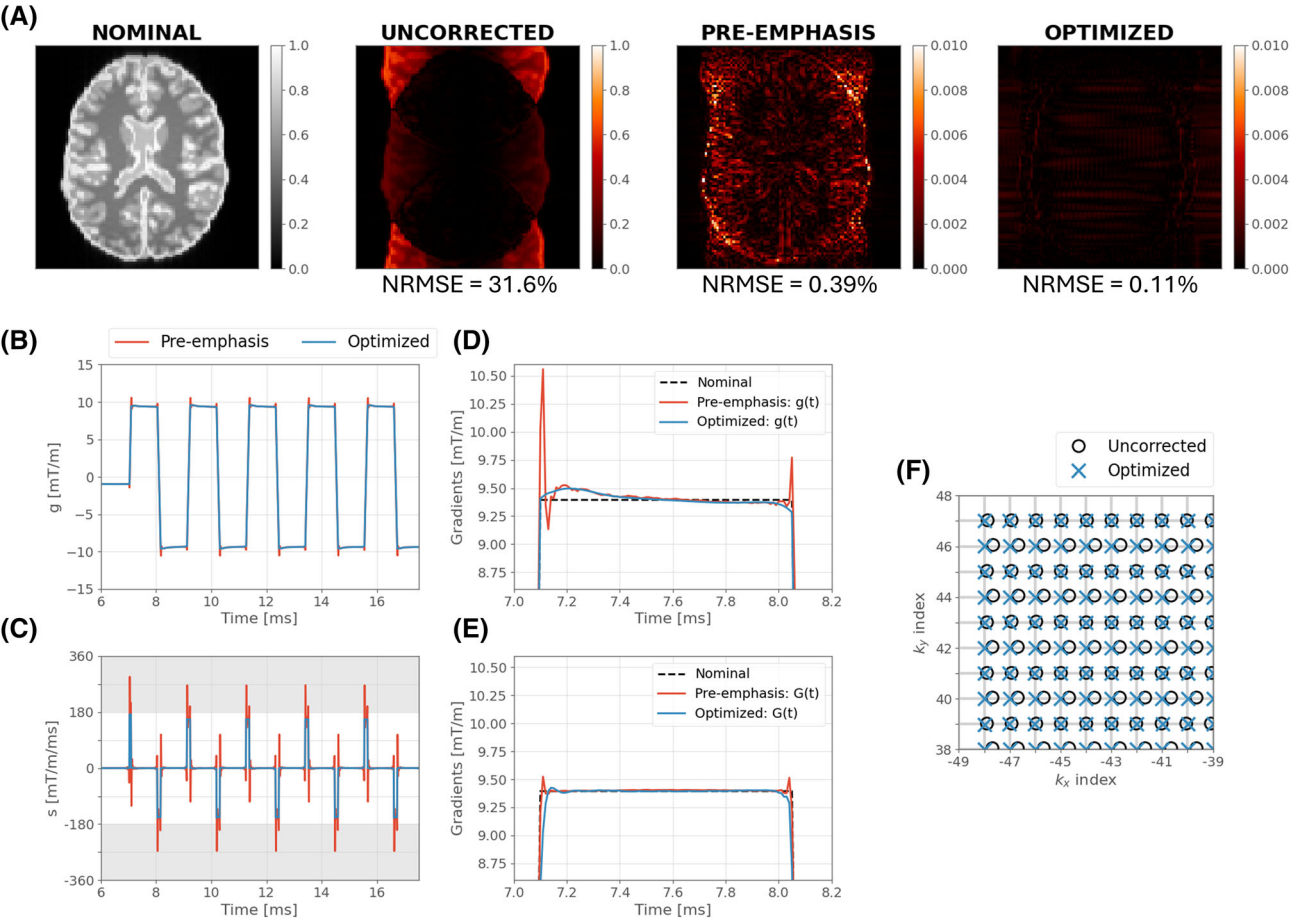
(A) Simulated images for a numerical brain phantom using the 7T gradient system transfer function (GSTF) and single‐shot (*R* = 1) echo‐planar imaging sequence. The nominal (target) image does not consider gradient imperfections, and the uncorrected image shows error predicted from GSTF without additional correction. GSTF‐based pre‐emphasis yields low error but exceeds the slew rate limit (180 mT/m/ms) (C). The right‐hand pane in (A) shows equivalent error from optimization, and its solution (blue demanded gradient traces in [B]) does not violate slew rate limits (C). Corresponding normalized root‐mean‐square error (NRMSE) is shown underneath each difference map. Zoomed optimized and GSTF‐based pre‐emphasis demanded (D) and realized (E) waveforms for one readout. The optimization solution remains within system limits and corrects k‐space locations (F).

Figure 3 illustrates simulated images for the single‐shot (*R* = 1) EPI sequence. When no correction is applied, images show significant ghosting artifacts. GSTF‐based pre‐emphasis yields a solution with low error (0.39%) that exceeds the slew rate limit. Optimization achieves a solution with lower error than pre‐emphasis (0.11%), while respecting gradient system constraints. This is achieved using subtle waveform changes that do not resemble the more dramatic “overdriven” pre‐emphasis solution (red line in Figure 3D). Note that pre‐emphasis using the simple exponential eddy current model (Figure 1A) returns a slightly lower error than our optimization, whereas in Figure 3, pre‐emphasis produces slightly higher error. Given that pre‐emphasis is not subject to any constraints, it might be expected that this would achieve lower error; for the empirical GSTF, this is not the case because of filtering used to limit noise from high frequencies in the GSTF. In both examples, the proposed optimization returns low error while also satisfying constraints.

### 4.3 Experimental results

**FIGURE 4:**
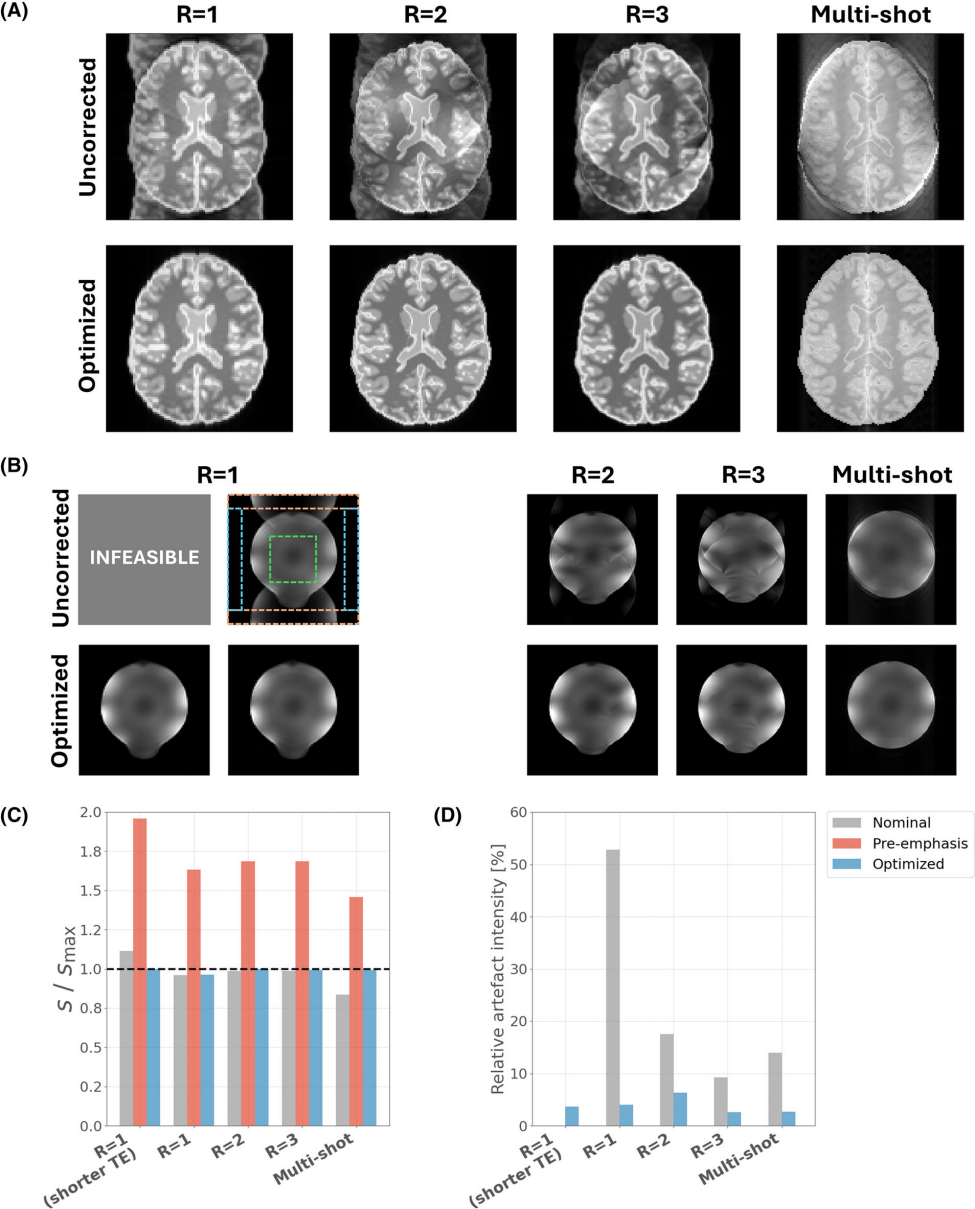
Simulated (A) and experimental (B) phantom results for echo‐planar imaging variants: *R* = 1, 2, and 3 and multishot using the 7T system. Simulated and experimentally acquired uncorrected sequences show image artifacts that are very similar in appearance. Optimization substantially reduces artifacts both in simulation and experiment. Experimental data contain two variants of the fully sampled (*R* = 1) case: one with shorter echo time (TE) (*left‐hand side*); this violates the slew rate limit, so an uncorrected image was not acquired, whereas optimization finds a solution that delivers this lower TE without ghosting. (C) Peak slew rate for each sequence, showing that our method always meets the specified constraint. (D) Relative artifact intensities computed from the outlined regions in (B) (difference between root mean square [RMS] signal in orange and blue boxes, normalized to RMS signal in green box). Pre‐emphasis using gradient system transfer function violates the slew rate limit for all cases, so was not tested experimentally.

Figure 4 depicts solutions for all EPI sequence variants described previously. Figure 4A displays simulated images using the numerical brain phantom (also used in Figures 1 and 3), whereas Figure 4B shows data acquired from the 7T system using a spherical water phantom. The phantom shape is slightly distorted due to local off‐resonance caused by the phantom holder, and signal shading is due to the receiver bias field that has been left uncorrected; neither is relevant to this discussion. Simulations (Figure 4A) predict significant ghosting in uncorrected image reconstructions for all variants that is eliminated by the optimization. The acquired phantom images show similar results, with uncorrected sequences yielding similar artifacts that are substantially reduced for the optimized sequences (indicated by decreases in relative artifact intensity in Figure 4D). Figure 4C shows that peak demanded slew rate always exceeds the limit ($s/s_{\max}$ = 1) using pre‐emphasis, so these solutions were not tested experimentally. Our method satisfies constraints for all scenarios, including the shorter TE sequence that could run using a shorter interecho spacing than possible with the nominal sequence.

**FIGURE 5:**
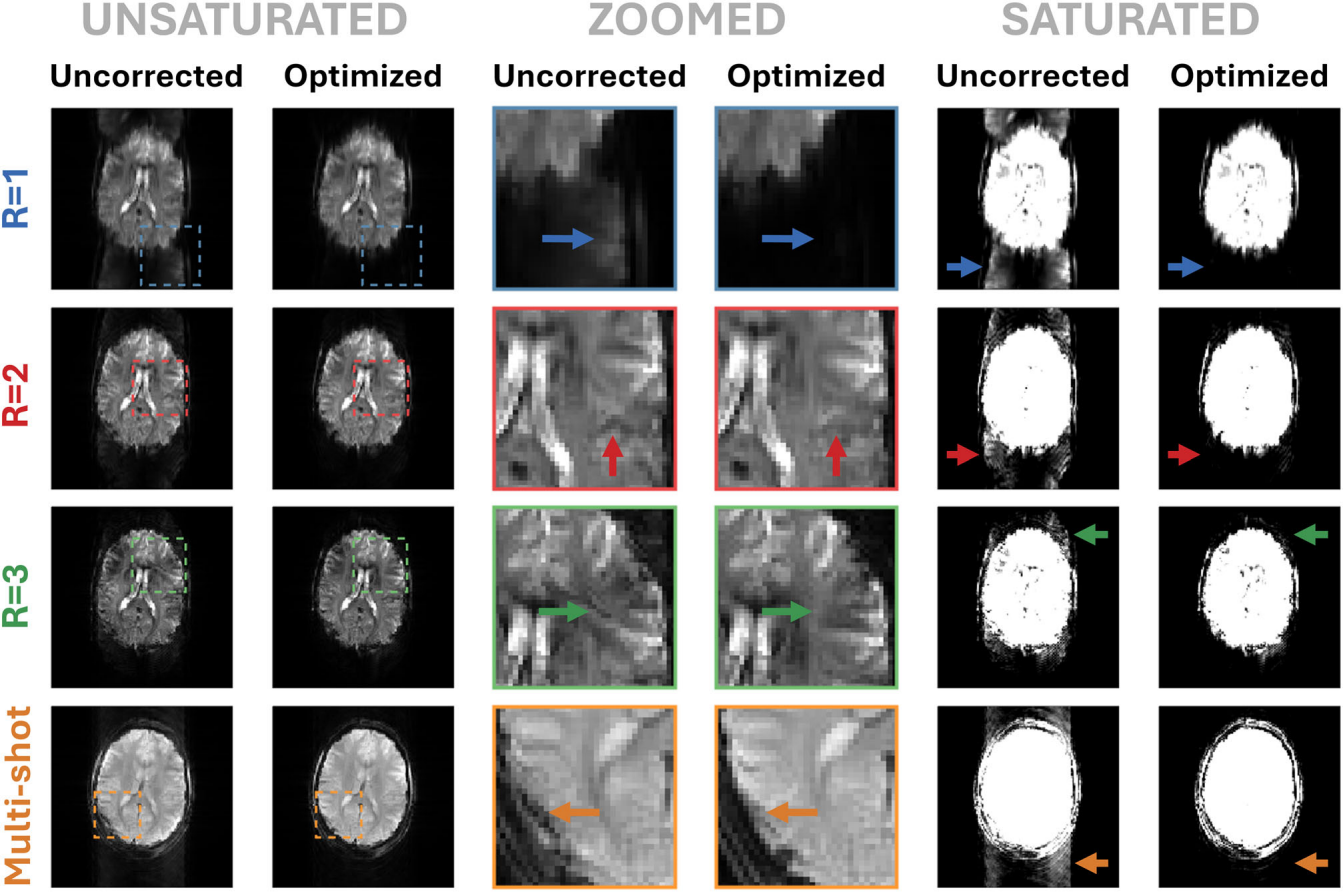
In vivo results for all echo‐planar imaging (EPI) variants. Ghosting artifacts inside and outside the brain are removed following optimization, as demonstrated by zoomed portions for each color‐coded EPI variant (*middle two columns*) and full‐image subplots with saturated color ranges (*two right‐most columns*). Color‐coded arrows indicate regions where the improvement with the proposed method is obvious. Identical color ranges are used for images acquired using corresponding uncorrected and optimized sequences.

The same sequences were tested in vivo with results presented in Figure 5 using different window settings to more clearly reveal the ghosts in uncorrected images. Ghosting is again substantially reduced for all variants, most notably for *R* = 1. Artifacts manifest themselves differently for multishot EPI but are also suppressed after optimization.

## 5 DISCUSSION AND CONCLUSIONS

This work proposes an optimization‐based method to design gradient waveforms, compensating for system imperfections via constrained optimization including an empirical model for gradient system performance. The approach differs from pre‐emphasis by (i) allowing amplitude/slew rate constraints to be respected and (ii) considering the achieved k‐space sampling, not the gradient waveform itself, during optimization. We explored use of a simple exponential eddy current model and a GSTF as the forward model, presenting both simulated images and experimental data that demonstrate image‐quality improvements using the proposed method. Because our approach requires optimization of sequences before scanning, it is unsuitable for existing system architectures where user‐defined resolution/fields of view dynamically change demanded gradients, requiring on‐the‐fly correction. Rather, this work is a proof of concept, where we explore the outlook and potential use cases below.

Figure 1 demonstrates an example for a short‐term (τ = 50 μs) eddy current, where classic pre‐emphasis (Eq. [2]) produces a large slew rate spike that cannot be achieved in practice. We demonstrate that numerical optimization can achieve correct k‐space sampling while respecting hardware limits. Moving to a real‐world example, Figures 2 and 3 focus on using the GSTF of a 7T system including all vendor‐implemented gradient corrections. Figure 2 depicts realized k‐space sampling from uncorrected sequences; errors are more complex than simple delays between adjacent lines. GSTF‐based pre‐emphasis^5^, ^6^, ^7^ can correct these deviations using frequency domain division, but as with the simpler eddy current model, resulting waveforms are likely to exceed physical constraints. The proposed method corrects trajectory‐related errors by directly optimizing gradient waveforms based on the achieved k‐space, rather than the distorted gradient waveforms themselves. The solutions found for several EPI sequences do not resemble the overdriven appearance of pre‐emphasis (e.g., Figure 3B–E), instead featuring more subtle modulations. The ability to work within constraints is a key benefit. Others have noted this limitation of pre‐emphasis, proposing to either “down‐rate” the initial waveforms^5^ (which compromises performance and is not optimal) or use iterative calculations with a time‐stretching algorithm to enforce physical constraints.^21^

This work is an acquisition‐based correction for imperfections. Reconstruction‐based, retrospective correction is also possible; indeed, simple phase correction of odd and even lines in an EPI acquisition is an example of this, as are more sophisticated methods using a GSTF^22^, ^23^, ^24^, ^25^ or temporal convolutional networks.^26^ In this proof‐of‐concept study, we only use iFT reconstruction (excluding any postacquisition correction methods), to focus on sampling fidelity. Ultimately, we envisage prospective and retrospective corrections working together. GSTF‐based optimization requires system stability to be effective. The success of our experimental work is testament to the fact that gradient systems in high‐performance scanners tend to be very stable, as noted by others who use GSTFs in image reconstruction.^22^, ^23^, ^24^, ^25^ There are data showing temperature‐dependence of the gradient response, but this effect can also be modeled.^27^ Although our optimizations were time‐consuming, the same solutions can be used in multiple experiments; phantom and in vivo data were obtained using identical gradient waveforms optimized with a GSTF acquired days earlier.

Others have also proposed gradient correction methods that are constrained by hardware limits. Harkins et al. explored predistortion of gradient waveforms to correct gradient errors in radiofrequency pulses^28^ and proposed an iterative correction scheme that includes repeated measurement of waveforms (useful when systems are less stable or not linear time‐invariant). The GrOpt toolbox^29^, ^30^ enables gradient waveform design under amplitude and slew rate constraints (among others). Applications have included individual preparation modules (e.g., diffusion‐encoding or velocity‐encoding gradients) that consider different properties of the gradient waveforms in the objective function. For diffusion, *b*‐value is the objective, whereas for phase‐contrast MRI, background phase error (due to the zeroth‐order moment) is the objective.^31^ Our work is related, as the k‐space objective function is also focused on the zeroth‐order moment of the designed waveforms, but we instead focus on whole EPI readouts rather than individual preparation modules.

The proposed optimization‐based approach must be performed for a specific field of view (including angulation, and for a fixed resolution). Modifying the waveforms retrospectively, using rotation matrices to rotate the field of view, for example, is not guaranteed to produce optimal results. This is very different from the current paradigm in MRI that uses corrections applied on‐the‐fly to enable maximum user flexibility. Future work will explore parameterized solutions that can be adapted to enable some user flexibility (e.g., field of view modification). Alternatively, future scanners could use fixed geometries to obtain acceptable performance from lower‐cost hardware.

We used the Adam optimizer, which is more typically used to train neural networks, as it was found empirically to work well (it is a gradient‐based method that performs well in high‐dimensional, nonconvex problems). Other optimizers will be explored in the future, as will a strategy that takes advantage of the time‐sequential nature of gradient waveforms. In terms of hardware models, we considered an analytic eddy current model, alongside an empirical GSTF that describes the response of the whole chain including GPAs and the controlling logic implemented on them (e.g., feedback control). We aim to develop more sophisticated models that include characteristics of the gradient power supply (e.g., voltage and charge capacity, thermal models). This approach may potentially allow hardware to be driven closer to its true limits, as will inclusion of other effects such as acoustic resonances and peripheral nerve stimulation.

## Supporting information


**Figure S1.** Gradient system transfer function (GSTF) first‐order self‐terms (*top*) and cross‐terms (*middle*). The latter are far smaller (note the change in y‐axis scale); however, the system model used for optimization includes all four terms for completeness. Zero‐order terms are displayed in the bottom subplot for reference but were not used in this work because their effect on the sequences studied was found to be small.

## Data Availability

The source code used for simulation studies and to generate sequences for in silico and in vivo experiments is available from https://github.com/mriphysics/Optimization‐for‐non‐ideal‐gradients.
