# Supplementary material for: MR sequence design to account for nonideal gradient performance
Source: Magn Reson Med. 2025 Sep 22;95(2):1135–45. doi: 10.1002/mrm.70093 (PMC12681289; doi:10.1002/mrm.70093)
Supplement: Supplementary file 1 — Figure S1. Gradient system transfer function (GSTF) first‐order self‐terms (top) and cross‐terms (middle). The latter are far smaller (note the change in y‐axis scale); however, the system model used for optimization includes all four terms for completeness. Zero‐order terms are displayed in the bottom subplot for reference but were not used in this work because their effect on the sequences studied was found to be small. [file MRM-95-1135-s001.docx]

**Supporting Information**


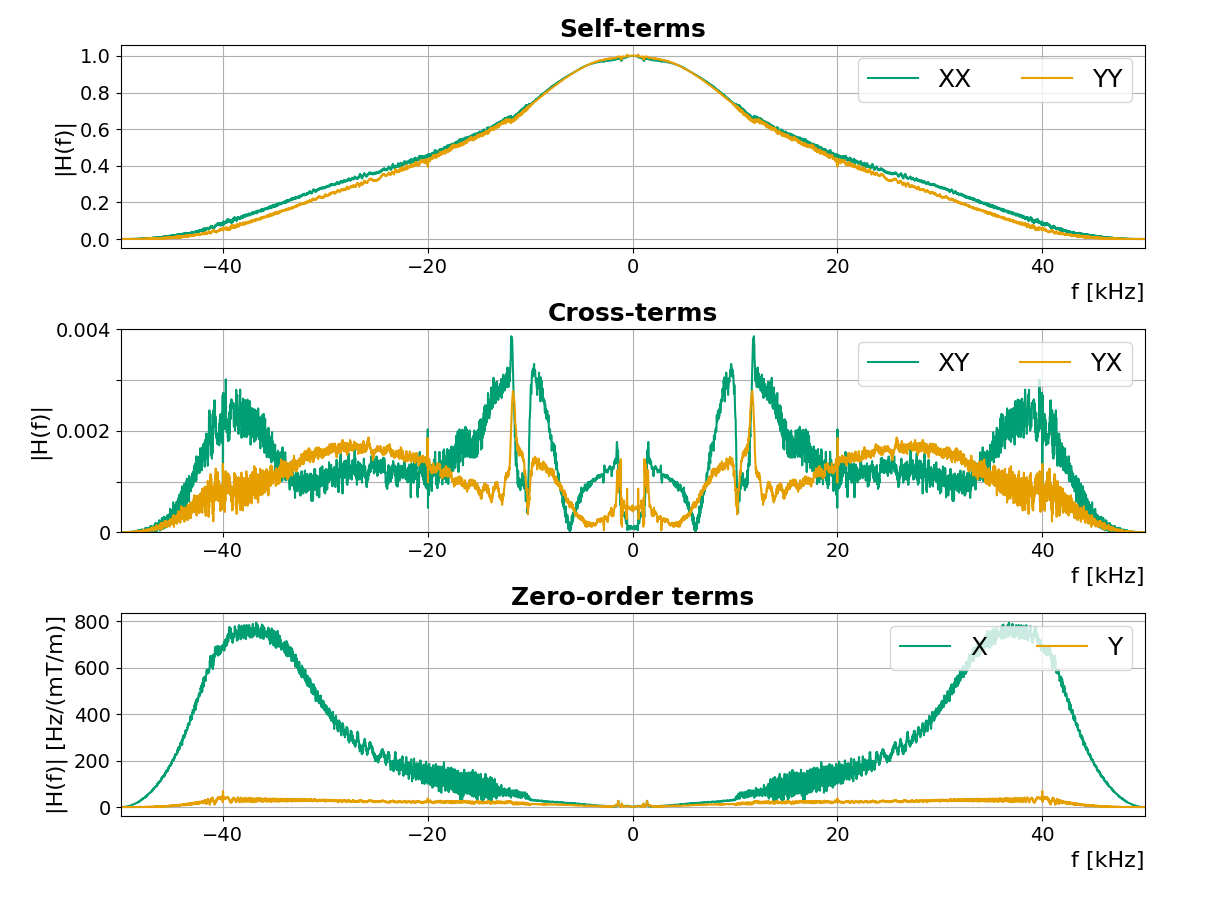


S1: GSTF first-order self-terms (top) and cross-terms (middle). The latter are far smaller (note the change in y-axis scale), however the system model used for optimization includes all four terms for completeness. Zero-order terms are displayed in the bottom sub-plot for reference but were not used in this work since their impact on the sequences studied was found to be small.
